# Novel fern- and centipede-like *Selaginella* (Selaginellaceae) species and a new combination from South America

**DOI:** 10.3897/phytokeys.91.21417

**Published:** 2017-11-28

**Authors:** Iván A. Valdespino

**Affiliations:** 1 Departamento de Botánica, Facultad de Ciencias Naturales, Exactas y Tecnología, Universidad de Panamá, Apartado Postal 0824-00073, Panama; 2 Sistema Nacional de Investigación (SNI), SENACYT, Panama

**Keywords:** Amazon basin, Guiana Highlands, New World, monomorphic, subgenera, tepuis, Cuenca del Amazonas, Escudo guyanés, monomórficas, Nuevo Mundo, subgéneros, tepuyes

## Abstract

Two new *Selaginella* species (i.e. *S. altheae* Valdespino and *S. squamulosa* Valdespino) and a novel combination [i.e. *S. philipsonii* (Jermy & Rankin) Valdespino] from South America are proposed. Descriptions, illustrations (line drawings and scanning electron micrographs, SEM, images), discussion on taxonomic affinities and information on habitat, distribution and phenology, as well as on conservation status are provided for each. *Selaginella altheae* is morphologically similar to species with erect, fern-like habit placed in the “*Selaginella flabellata* (L.) Spring group” as defined by Hieronymus, while *S. squamulosa* is allied to a species assembly with centipede-like habit here informally termed the “*Selaginella vernicosa* Baker group;” whereas *S. philipsonii* with its moss-like habit may be associated with species in the “*Selaginella jungermannioides* (Gaudich.) Spring group” or those centered on *S. ovifolia* Baker. All taxa here proposed are classified in subg. *Stachygynandrum*.

## Abstract (es)

Dos nuevas especies de *Selaginella* (i.e., *S. altheae* Valdespino y *S. squamulosa* Valdespino) y una nueva combinación [i.e., *S. philipsonii* (Jermy & Rankin) Valdespino de Sur América son propuestas. Para cada una de ellas se provee una descripción, ilustraciones (i.e. dibujos y micrografías de microscopio electrónico de barrido, MEB), discusión sobre su afinidad taxonómica, información sobre su hábitat, distribución y fenología, así como su estado de conservación. *Selaginella altheae* es morfológicamente similar a las especies con hábito erecto y parecidas a los helechos que se incluyen en el “grupo de *Selaginella flabellata* (L.) Spring” como fue definido por Hieronymus; *S. squamulosa* es afín a especies con hábito centipediforme que aquí informalmente se denominan “grupo de *Selaginella vernicosa* Baker”, mientras que *S. philipsonii* con su hábito parecido al de los musgos puede estar emparentada con el “grupo de *Selaginella jungermannioides* (Gaudich.) Spring” o a las especies similares a *S. ovifolia* Baker. Por último, todos los taxa aquí propuestos se clasifican en el subg. *Stachygynandrum*.

## Introduction

Continued work on South American *Selaginella* leads to the description of two new taxa: *Selaginella altheae* Valdespino and *S. squamulosa* Valdespino and to raise to species level *S. philipsonii* (Jermy & Rankin) Valdespino. These three species are anisophyllous either throughout the stems or shortly below or after the first branches with the leaves arranged in four (i.e. two median and two lateral) distinct ranks and quadrangular strobili composed of monomorphic sporophylls. Accordingly, they can be placed within subg. *Stachygynandrum* (P. Beauv.) Baker following Jermy’s (1986, 1990) morphology-based classification or that of Weststrand and Korall (2016) supported by phylogenetic analysis using molecular and morphological characters. Alternatively, they could each be assigned to two or three subgenera (i.e. *Ericetorum*, *Stachygynandrum* and *Heterostachys*) proposed by Zhou and Zhang (2015). It is believed, however, that placement of these three species in subg. *Stachygynandrum* best reflects their morphology, allows for easy identification and facilitates comparison with other taxa.

*Selaginella altheae* has a fern-like habit with erect main stems and axillary, ventral, dorsal and, occasionally, lateral rhizophores. Morphologically, this species belongs to the “*Selaginella flabellata* (L.) Spring group” as defined by Hieronymus (1901: 682), which contains ca. thirty-five species in the Americas (Valdespino, unpublished). *Selaginella squamulosa* seems morphologically related to a species group comprising about seven taxa including *S. arrecta* A.R. Sm., *S. marahuacae* A.R. Sm., *S. roraimensis* Baker, *S. scalariformis* A.C. Sm. and *S. vernicosa* Baker and the newly described *S. psittacorhyncha* Valdespino (Valdespino 2017), which together are herein informally termed the “*Selaginella vernicosa* Baker group”. This group is defined by its centipede-like habit (i.e. plant body elongated and flattened, branching in one plane with leaves suggestive of body segments; overall reminiscent of centipedes), creeping or ascending to suberect stems and coriaceous leaves. *Selaginella philipsonii* has a moss-like habit (i.e. resembling a pleurocarpous moss in its long-creeping main stems and short lateral branches that often bear reproductive structures), axillary and dorsal rhizophores and small leaves. It may be related to species in the “*Selaginella jungermannioides* (Gaudich.) Spring group”, as circumscribed in Valdespino et al. (2015), or to a species group akin to *S. ovifolia* Baker.

These additions to Neotropical *Selaginella* yield the 100 species estimated by Valdespino (2015, 2016) for Venezuela and raises the number of native Brazilian taxa to 84, including *S. psittacorhyncha* and the recently recorded *S. anaclasta* Alston ex Crabbe & Jermy in Serra do Aracá, Brazil (Barbosa-Silva et al. 2016), based on *Labiak et al*. *5639* (NY!). Thus, Brazil is now the country with the second highest *Selaginella* diversity in the New World. In the case of Colombia, more studies are still needed to ascertain its *Selaginella* diversity. It is likely that the number of Colombian species will be greater than that reported by Alston et al. (1981).

## Material and methods

Herbarium studies were carried out by examining specimens from BM, CAS, COL, F, GH, MO, NY, OXF, P, PMA, RB, UC, US and W, as well as digitized images from COL and RB (herbarium acronyms follow Thiers 2017). Scanning electron microscopy (SEM) micrographs were made from selected specimens to document leaf surfaces and mega- and microspores sculpturing patterns, when available, from designated types and paratypes. These studies were conducted according to standard techniques as described by Valdespino (1995) and viewed and digitized at different magnifications using a Zeiss Model Evo 40 SEM at 10–20 kV at the Smithsonian Tropical Research Institute (STRI) in Panama. Digitized SEM images were post-processed and assembled in multipart figures using Adobe Photoshop as explained in Valdespino (2016).

Terminology and measurements of leaves and spores, as well as species conservation status provided in descriptions were assessed following Valdespino (2016 and references therein). Countries, in the additionally examined specimens section, were cited according to geographical order.

## Taxonomy

### Selaginella altheae

Taxon classificationPlantaeSelaginellalesSelaginellaceae

Valdespino sp. nov.

urn:lsid:ipni.org:names:77173690-1

**Figure 1.**
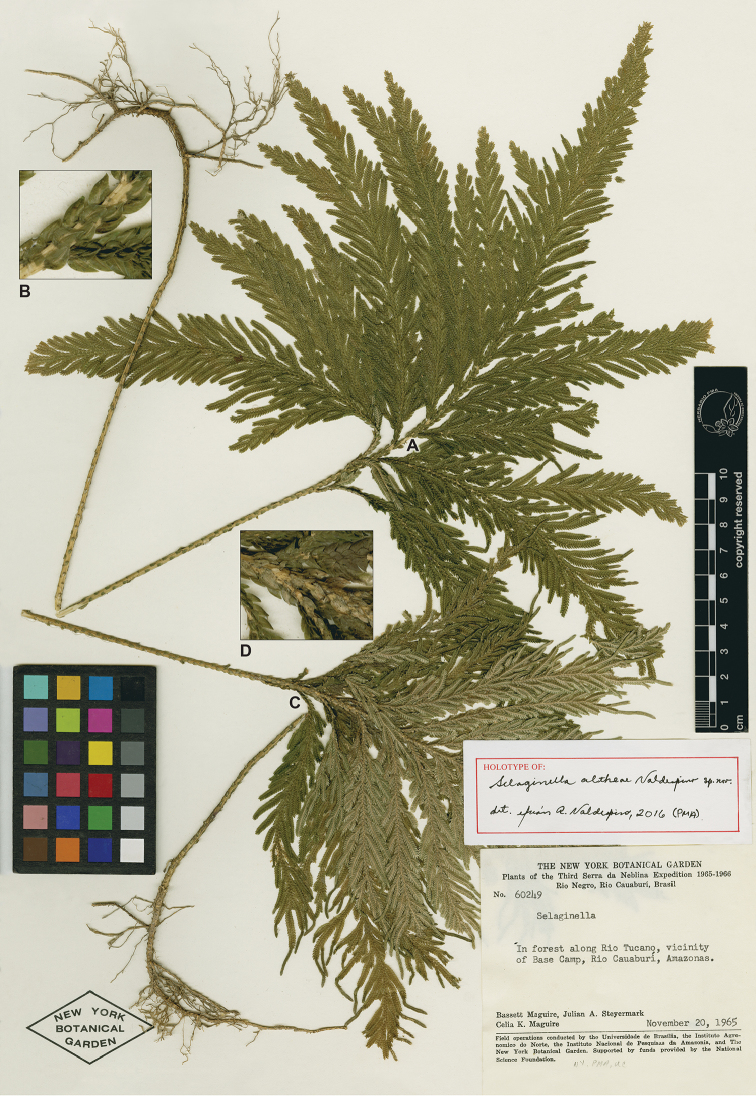
*Selaginella altheae* Valdespino. **A** Habit, upper surface of stem **B** Close up of section of the upper surface of the stem **C** Habit, lower surface of stem **D** Close up of section of the lower surface of the stem. **A–D** digitized images of holotype, *Maguire et al. 60249* (NY).

**Figure 2.**
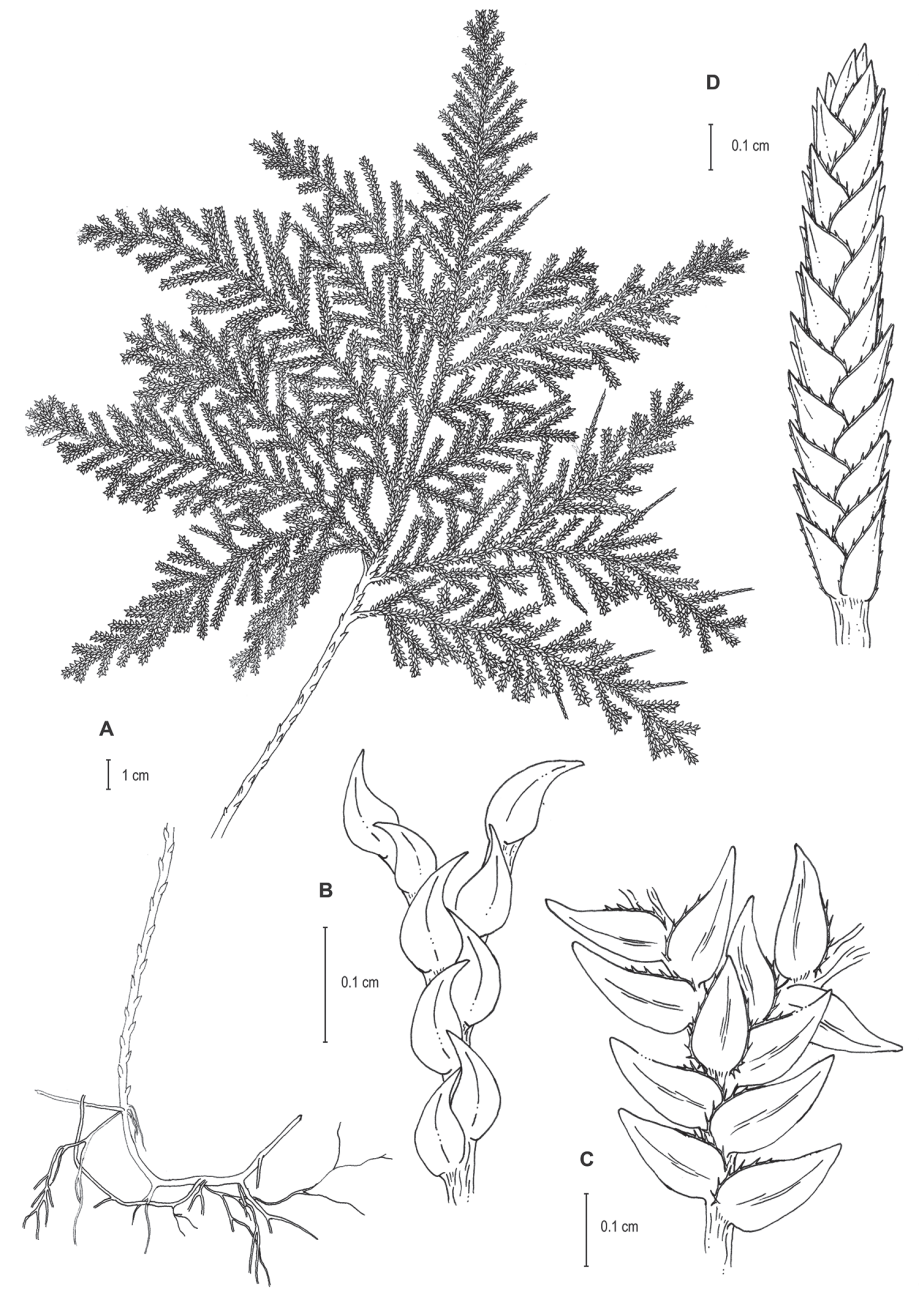
*Selaginella altheae* Valdespino. **A** Habit **B** Section of main stem above first branch showing median leaves, upper surface **C** Section of main stem above first branch showing lateral leaves and axillary leaf, lower surface **D** Close-up of strobilus. **A–D** line drawings of holotype, *Maguire et al. 60249* (NY). Illustration made by Rubén Lozano.

**Figure 3.**
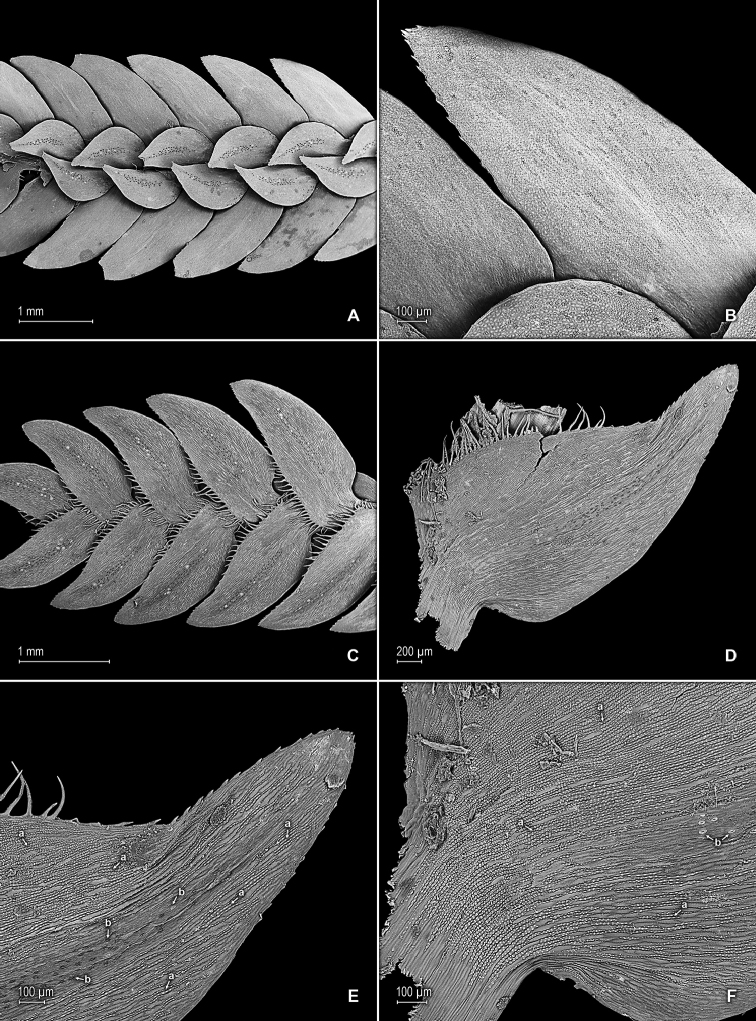
*Selaginella altheae* Valdespino. **A** Section of upper surface of stem branch **B** Close-up of lateral leaf from stem branch, upper surface **C** Section of lower surface of stem branch **D** Close-up of lateral leaf (from branch), lower surface **E** Close-up of distal portion and apex of lateral leaf, lower surface (same leaf shown in **B**); note, elongate and papillate idioblasts (a) and stomata along midrib (b) **F** Close-up of proximal (basal) portion of lateral leaf, lower surface (same leaf shown in **B**); note, elongate and papillate idioblasts (a) and stomata along midrib (b). **A–F** taken from holotype, *Maguire et al. 60249* (NY).

**Figure 4.**
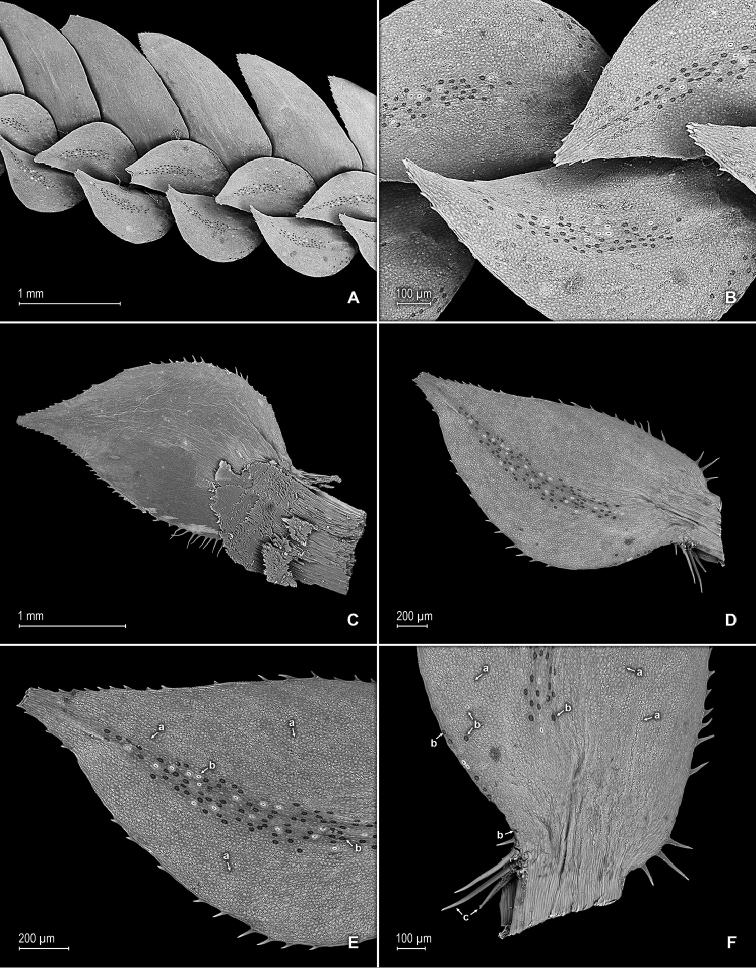
*Selaginella altheae* Valdespino. **A** Section of upper surface of stem branch showing median and lateral leaves **B** Close-up of median leaves from stem branch, upper surfaces **C** Close-up of median leaf from stem branch, lower surface **D** Close-up of median leaf from stem branch, upper surface **E** Close-up of distal portion and apex of median leaf, upper surface (same leaf shown in **D**); note, punctate to shortly elongate and papillate idioblasts (a) and stomata (b) along midrib **F** Close-up of proximal portion and base of median leaf, lower surface (same leaf shown in **D**); note, punctate to shortly elongate and papillate idioblasts (a), stomata (b) along midrib and submarginal and marginal portion near outer base and long cilia (c) on outer base. **A–F** taken from holotype, *Maguire et al. 60249* (NY).

**Figure 5.**
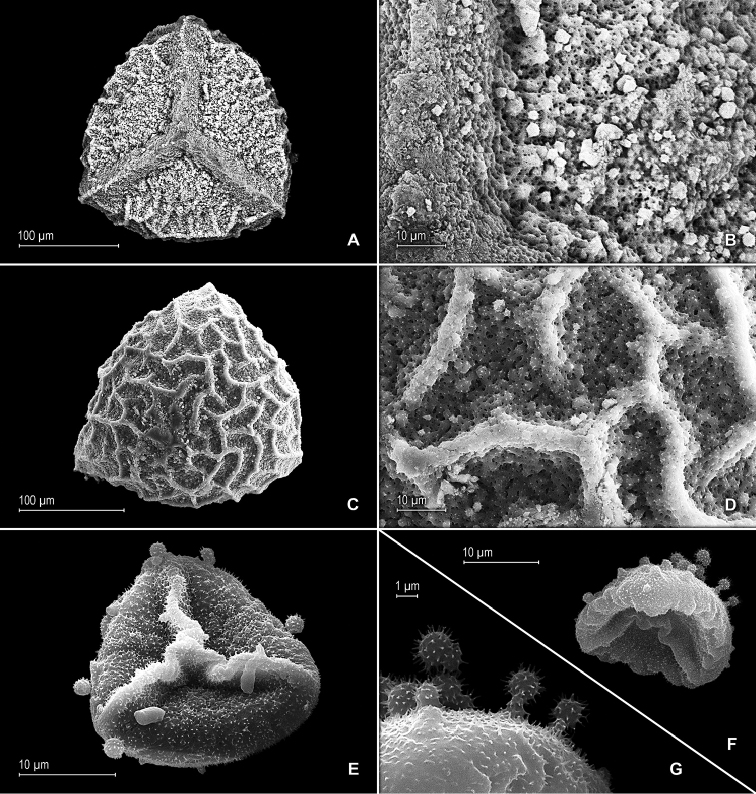
*Selaginella altheae* Valdespino. **A** Megaspore, proximal face **B** Close-up of megaspore, proximal face **C** Megaspore, distal face **D** Close-up of megaspore, distal face **E** Microspore, proximal face **F** Microspore, distal-equatorial-proximal faces **G** Close-up of microspore, distal-equatorial faces; note, capitate projections and echinulate microstructure. **A–G** taken from holotype, *Maguire et al. 60249* (NY).

Figures 1 , 2 , 3 , 4 , 5

#### Diagnosis.

*Selaginella altheae* differs from *S. lechleri* Hieron. by the main stem leaves becoming obviously dimorphic above or 1–4 cm (vs. 3–6 cm) below the first branches of the stems, its lateral leaves bases oblique with a seemingly inner [actually it is an acroscopic] (vs. lacking) auricle that is tufted with 3–15 hairs (vs. bases glabrous) and the axillary leaves rounded to cordate or truncate with two, distinct, incurved and ciliate (vs. lacking) auricles at least up to the third or fourth branches along the stems.

#### Type.

**BRAZIL**. Amazonas: Río Negro, Río Cuaburí, along Río Tucano, vicinity of Base Camp, 20 Nov 1965, *B. Maguire, J.A. Steyermark & C.K. Maguire 60249* (holotype: NY).

#### Description.

*Plants* terrestrial. *Stems* erect, stramineous, (20)30–65 cm long, 1.4–3.0 mm diam., non-articulate, usually not flagelliform, stoloniferous, 2–4-branched. *Rhizophores* axillary, ventral and dorsal, borne on lower-most part of the stems and throughout stolons, filiform or stout, 0.2–1.0 mm diam. *Leaves* seemingly monomorphic and strongly appressed to the stem shortly before or after first branches, then dimorphic throughout, coriaceous, upper surfaces dull to shiny green, slightly corrugate, lower surfaces shiny yellowish green to silvery green, striate, the outer bases short- to long-auricled (these tend to disappear on leaves above first branches), the auricle short- to long-ciliate, cilia 2–10, each 0.2–0.5 mm long. *Lateral leaves* on main stem after first branches distant to slightly imbricate, strongly ascending to slightly spreading, ovate-deltate to ovate-oblong, 2.0–4.2 × 0.9–2.2 mm; bases truncate, on main stem before first branches with an inner, acroscopic bases strongly overlapping stems, rounded, entire, basiscopic bases free from stems, geniculate, usually ciliate, cilia 1–5, each 0.1 to 0.2 mm long; acroscopic margins on main stem leaves after second branches greenish to narrowly hyaline along proximal ⅔, 1–3 cells wide with the cells elongate, sinuate-walled and glabrous, parallel to margins, otherwise greenish, on branch leaves narrowly hyaline along proximal ¾ and cells as those on main stem, otherwise greenish distally, long-ciliate along proximal ½–¾, otherwise becoming short-ciliate to dentate distally, basiscopic margins greenish, comprising similar cells as in acroscopic margins, entire along proximal ⅔, otherwise sparsely denticulate distally; apices gradually tapering, acute to broadly acute, tipped by 1–3 teeth; upper surfaces comprising irregularly shaped, somewhat rectangular to quadrangular, sinuate-walled cells (often difficult to distinguish because of waxy deposits), with some obscure, idioblast-like, short quadrangular to round or elongate and variously papillate cells, without stomata or with some obscure along basiscopic margins, lower surfaces comprising elongate, sinuate-walled cells, with many of these idioblast-like, elongate and papillate, papillae 5–14 in 1 or 2 rows on each cell lumen, stomata on 1–5 rows along midribs. *Median leaves* on main stems after first branches distant to slightly imbricate, ascending, ovate, broadly ovate to ovate-elliptic, 1.4–3.0 × 0.8–1.7 mm; bases truncate to truncate-oblique, the outer base tufted with (4)6–20 long hairs, without auricles; margins greenish to narrowly hyaline, 1 or 2 cells wide with the cells elongate, slightly sinuate-walled and mostly glabrous or papillate, parallel to margins, the inner margins short-ciliate along proximal ⅔, denticulate along distal ⅓, the outer margins entire along proximal ⅓, becoming short-ciliate along medial ⅓, otherwise denticulate on distal ⅓; apices acute to slightly acuminate, the acumen ca. 0.1 mm, tipped by 1–3 teeth; upper surfaces comprising irregular (jigsaw puzzle-like), sinuate-walled cells (often difficult to distinguish because of waxy deposits), some of these papillate, papillae 3–14, irregularly arranged, without elongate idioblasts, stomata in 3–7 rows along the midribs and marginal to submarginal along basiscopic ⅓ of outer margins, lower surfaces comprising elongate (jigsaw puzzle-like), sinuate-walled cells, without elongate idioblasts and stomata. *Axillary leaves* on main stem after first branches ovate-lanceolate, 2.0–3.0 × 0.9–1.5 mm; bases rounded to cordate or truncate, with two small, long-ciliate auricles up to, at least, the fifth branch and then becoming small lobes; margins narrowly hyaline as in median leaves, long-ciliate along proximal ⅘, otherwise short-ciliate to denticulate distally; apices gradually tapering, acute, tipped by 1–3 teeth; both surfaces as lateral leaves. *Strobili* terminal on main stem and each branch tip, quadrangular, 0.3–1.7 cm. *Sporophylls* monomorphic, without a laminar flap, each with a slightly developed and seemingly glabrous keel along midribs, ovate to ovate-lanceolate, 0.8–1.0 × 0.4–0.6 mm; bases rounded to truncate; margins narrowly hyaline, 1 or 2 cells wide with the cells elongate, slightly sinuate-walled and glabrous, parallel to margins, short-ciliate along proximal ½, otherwise denticulate to entire distally; apices acute to acuminate, the acumen 0.1 to 0.2 mm, tipped by 3 tooth-like projections; *dorsal sporophylls* with upper and lower surfaces as in vegetative leaves; *ventral sporophylls* with both surfaces, silvery green to hyaline, comprising elongate, papillate, sinuate-walled cells. *Megasporangia* in 2 ventral rows; *megaspores* white, rugulate-reticulate on proximal faces with a slightly developed equatorial flange and microstructure granulate, perforate and sparsely echinate, mostly open to somewhat closely reticulate on distal faces with the microstructure sparse and minutely echinate and perforate, 180–250 µm diam. *Microsporangia* in 2 dorsal rows; *microspores* orange, echinate-rugulate on proximal faces with punctate microstructure, capitate or baculate on distal faces with each caput or bacula and the rest of the surface with echinate microstructure, 20–30 µm diam.

#### Habitat, distribution and phenology.

*Selaginella altheae* grows on lowland and montane rainforests at 140–450 m; it is known from the Amazon basin of Colombia, Venezuela and Brazil and found fertile from April through to November.

#### Eponomy.

I am delighted to dedicate this graceful, fern-like species with shiny idioblasts on leaf surfaces to my mother, Althea Quintero (1939–), whose gentle, selfless demeanor, as well as strength and enlightening life-long support has guided and buttressed my life and professional career. She has adorned my path in life in the same manner as her lovely and resilient namesake flower does in nature.

#### Conservation status.

This is a widely distributed species that grows at low elevations in Tropical rainforests of the Amazon basin; therefore, it is considered here of Least Concern (LC), according to IUCN (2012) categories and criteria.

#### Additional specimens examined (paratypes).

**COLOMBIA**. **Amazonas**: Río Amazonas, along a road 8–14 km N of Leticia, 450 m, 3–5 Jul 1974, *Breedlove 36331* (CAS).**VENEZUELA**. **Amazonas**: Rivers Casiquiare, Vasiva [Pasiba] and Pasimoni, 1853–54, *Spruce 3380* (GH, OXF, W); Depto. Río Negro, along river that flows out of the Canyon Grande of Cerro de la Neblina, 2–4 km upriver from base, 10 Feb 1984, *Funk & Liesner 6148* (NY, RB [digital image], US), trail leading N-NE, 28 Feb 1984, *Funk 6419* (MO, NY, US), Río Mawarinuma, 00°50'N, 66°10'W, 140 m, 27 Nov 1984, *Anderson 13372* (CAS, F, UC), Cerro de la Neblina Base Camp on Río Bario (Río Mawarinuma), SE of camp, 00°49'50"N, 66°09'40"W, 140 m, 26 Jan 1985, *Beitel & Buck 85042* (NY), 27 Jan 1985, *Beitel & Buck 85064* (MO, NY, UC), *Beitel & Buck 85068* (NY), 17 Feb 1985, *Beitel & Buck 85210* (NY-2 sheets), 4 Dec 1984, *Bell 406* (UC), 2–3 Jul 1984, 00°50'N, 66°10'W, 25 Nov 1984, *Croat 59312* (MO, NY), 2–3 Jul 1984, *Davidse & Miller 26886* (UC), 4–5 Jul 1984, *Davidse & Miller 26972* (MO, UC), ca. 160 m, 27 Nov 1984, *Kral & Liesner 71851* (UC), ca. 180 m, 4 Dec 1984, *Kral 71997* (UC), 0 to 2 km W of Cerro de La Neblina Base Camp, which is on Río Mawarinuma, 140 m, 00°50'N, 66°10'W, 7 Feb 1984, *Liesner 15716* (MO), Río Mawarinuma, below Cerro Neblina Camp, 00°50'N, 66°11'W, 140 m, 16 Apr 1984, *Gentry & Stein 46677* (MO), ca. 2 km S of Base Camp [Cerro Neblina base camp], along Marawanumi River, 00°50'N, 66°10'W, 140 m, 11 Apr 1984, *Thomas & Plowman 3014* (NY), along Río Marawinuma, SE of Base Camp [Cerro Neblina base camp], 00°50'N, 66°09'W, 140 m, 30 Apr 1984, *Thomas & Samuels 3291* (NY, PMA).

#### Discussion.

*Selaginella altheae* is characterized by its erect, fern-like habit, axillary, lateral and dorsal rhizophores, leaves on main stems becoming obviously dimorphic above or 1–4 cm below the first branches and, below this point, the lateral leaf bases oblique with an acroscopic auricle that is tufted with 3–15 stiff hairs and median leaves above first branches in main stems with oblique, not auricled bases, with the outer bases slightly prominent and tufted with (4)6–20stiff hairs.

*Selaginella altheae*, *S. lechleri* and other members of the *S. flabellata* group, have microspores distal faces with echinate microstructure. Likewise, *S. altheae* and *S. oaxacana* Spring have dorsal rhizophores, as do other members of the *S. flabellata* group. These two characters, therefore, might represent synapomorphies that define the *S. flabellata* group. Nevertheless, dorsal rhizophores, which are typical of articulate *Selaginella* species, are also found in other taxa such as *S. psittacorhyncha* (Valdespino 2017) and *S. philipsonii*, suggesting that this character is under-reported and, perhaps, of a wider presence in the genus than hitherto acknowledged. Similarly, different degrees of spore echinate microstructure could occur in other unrelated taxa. Therefore, the occurrence of these characters in morphologically unrelated species warrants further morphological, anatomical and molecular studies throughout the genus to ascertain their evolutionary and phylogenetic implications. Interestingly, a specimen of *S. altheae* (i.e. *Thomas & Samuels 3291*, NY) has flagelliform tertiary branch apices that develop strobilus at their tips.

*Selaginella altheae* is morphologically close to *S. flabellata* and *S. lechleri*. Their leaves on the main stems below the first branches are seemingly monomorphic and have similar median leaf above the first branches with the outer bases slightly prominent and tufted with either few or many short- to long cilia, as well as submarginal to marginal stomata along the proximal ⅓ on each outer half of the laminae. Nevertheless, *S. altheae* is set aside from *S. flabellata* by its median leaves on main stems ovate, broadly ovate to ovate-elliptic (vs. broadly ovate to ovate-oblong) with the inner and outer halves on the main stems equal in width or the inner halves slightly wider than the outer halves (vs. usually the outer halves distinctly wider) and lateral leaves acroscopic margins long-ciliate along proximal ½–¾ (vs. ¼–½), otherwise distally short-ciliate to dentate (vs. entire or denticulate). It differs further from *S. flabellata* by its megaspores proximal faces rugulate-reticulate (vs. rugulate) with granulate, perforate and sparsely echinate and perforate (vs. tuberculate, mostly psilate to minutely echinate and perforate) microstructures and distal faces mostly open or somewhat closely reticulate (vs. mostly closely reticulate) with minutely echinate and perforate (vs. prominently tuberculate, psilate and perforate) microstructures. *Selaginella altheae* differs from *S. lechleri*, with which it has been confused in the past, by the characters discussed under the diagnosis and by the megaspores proximal faces rugulate-reticulate (vs. reticulate) with granulate, perforate and sparsely echinate (vs. with strongly echinate and perforate) microstructures, while the distal faces are mostly open or somewhat closely reticulate (vs. mostly closely reticulate) with sparse and minutely echinate and perforate (vs. abundantly echinate and perforate) microstructures.

*Selaginella altheae* may also be confused with the Central and South American *S. anceps* (C. Presl) C. Presl because of their auriculate leaf bases (i.e. auriculae present on lateral leaves of *S. altheae* and on seemingly monomorphic leaves of *S. anceps*) on main stems below the first branches. *Selaginella altheae* is set aside from the latter by its obviously dimorphic leaves immediately above or 1–4 cm below the first (vs. above fourth) branches of the main stems and median leaf bases on main stems above the first branches without auricles (vs. with inner and outer ciliate auricles or only with an outer ciliate auricle). It is further distinguished from *S. anceps* by its megaspores proximal faces with a slightly developed (vs. lacking) equatorial flange, rugulate-reticulate (vs. reticulate) ornamentation with granulate, perforate and sparsely echinate (vs. with perforate and strongly echinate) microstructures, while the distal faces ornamentation is mostly open or somewhat (vs. mostly) closely reticulate with sparse and minutely echinate and perforate (vs. shortly echinate, granulose and perforate) microstructures.

### Selaginella squamulosa

Taxon classificationPlantaeSelaginellalesSelaginellaceae

Valdespino sp. nov.

urn:lsid:ipni.org:names:77173691-1

**Figure 6.**
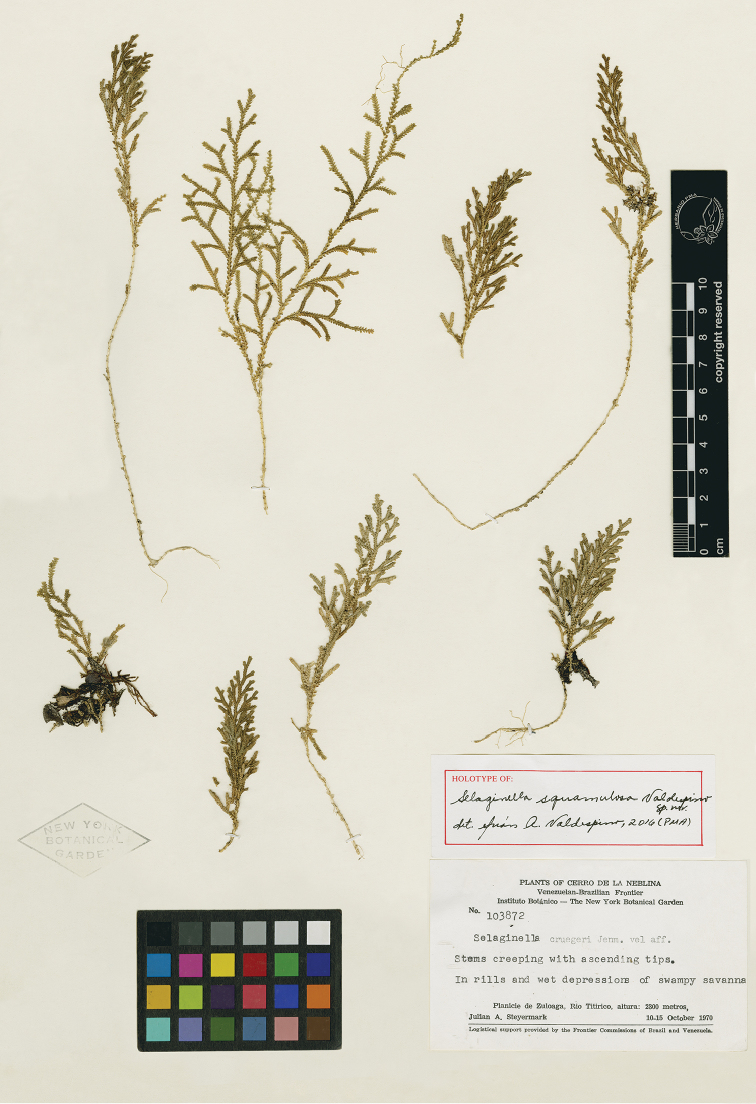
*Selaginella squamulosa* Valdespino. Holotype, *Steyermark 103872* (NY).

**Figure 7.**
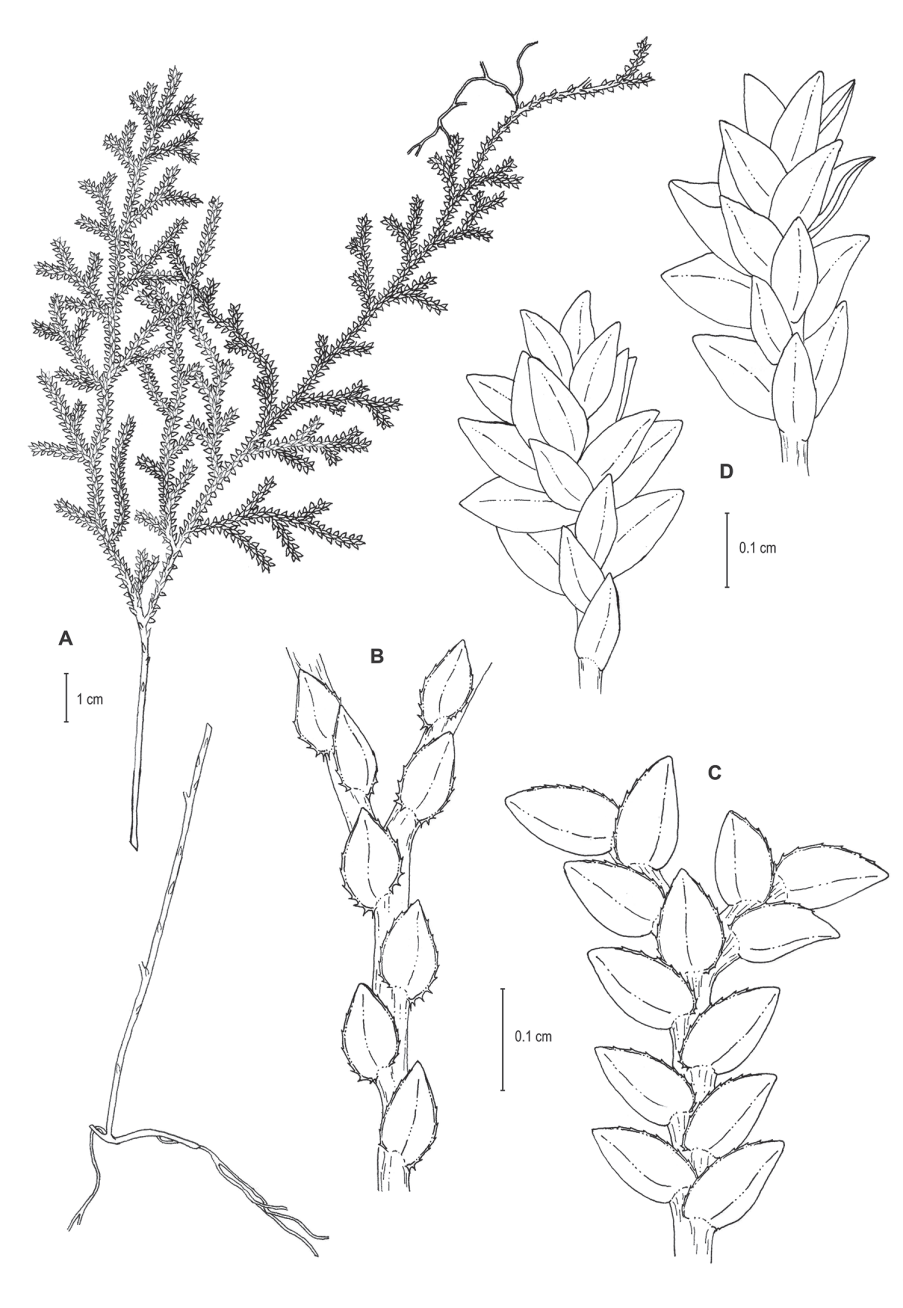
*Selaginella squamulosa* Valdespino. **A** Habit **B** Upper surface of stem showing median leaves **C** Lower surface of stem showing lateral leaves and axillary leaf **D** Close-up of strobili (apices of branches), upper surface. **A–D** line drawings of holotype, *Steyermark 103872* (NY). Illustration made by Rubén Lozano.

**Figure 8.**
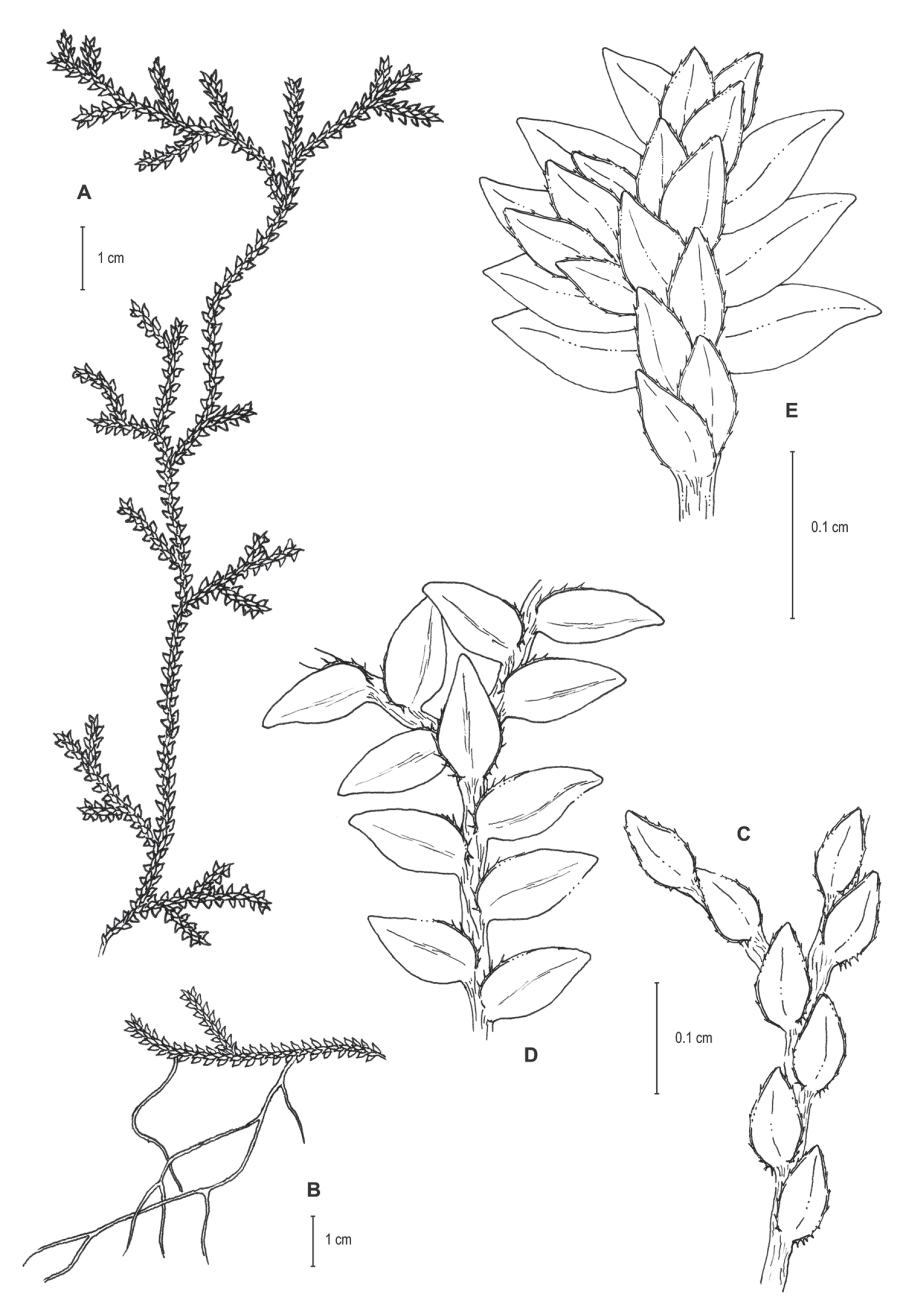
*Selaginella squamulosa* Valdespino. **A–B** Habit **C** Upper surface of stem showing median leaves **D** Lower surface of stem showing lateral leaves and axillary leaf **E** Close-up of distal portion of stem, upper surface. **A–E** line drawings of paratype, *Boom et al. 6011* (NY). Illustration made by Rubén Lozano.

**Figure 9.**
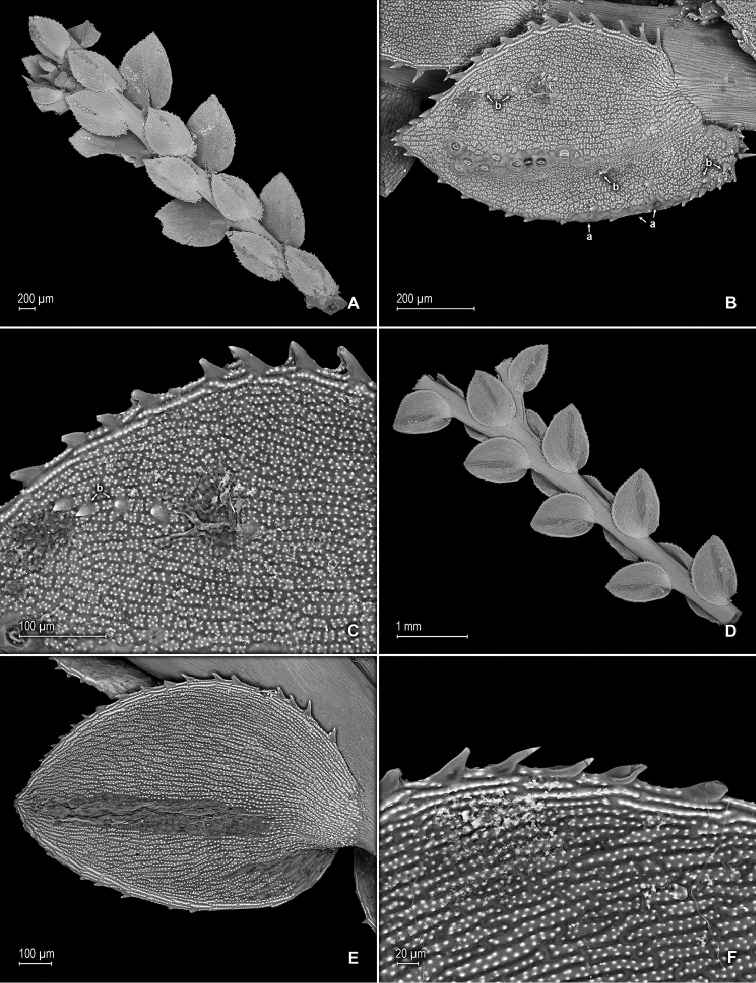
*Selaginella squamulosa* Valdespino. **A** Section of upper surface of stem **B** Close-up of median leaf, upper surface; note, elongate, idioblast-like marginal cells on inner margin and submarginal and marginal stomata (a) along proximal ½ of outer margin and hairs (b) on lamina surface **C** Close-up of mid-distal portion of median leaf, upper surface (same leaf shown in B); note, elongate, idioblast-like marginal cells on inner margin, papillae on each cell lumen and hairs (b) on lamina surface **D** Section of lower surface of stem **E** Close-up of lateral leaf, lower surface **F** Close-up of section of acroscopic half of lateral leaf; note, papillae on cells lumen and marginal, idioblast-like and papillate cells. **A–F** taken from paratype, *Boom et al. 6011* (NY).

**Figure 10.**
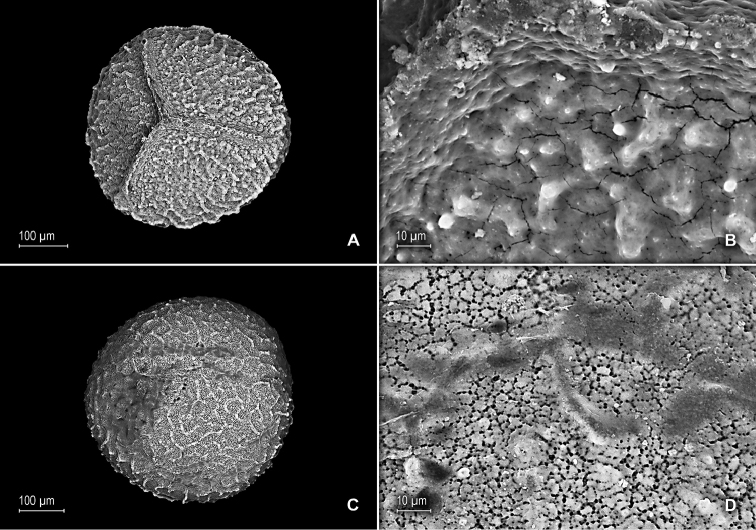
*Selaginella squamulosa* Valdespino. **A** Megaspore, proximal face **B** Close-up of megaspore, proximal face **C** Megaspore, distal face **D** Close-up of megaspore, distal face. **A–D** taken from paratype, *Boom et al. 6011* (NY).

Figures 6 , 7 , 8 , 9 , 10

#### Diagnosis.

*Selaginella squamulosa* is set aside from similar *S. psittacorhyncha* and *S. vernicosa* by its median leaves upper surfaces with 10 (vs. with 25 in *S. psittacorhyncha* and 35 in *S. vernicosa*) stomata along the midribs. It differs further from *S. psittacorhyncha* by its lateral leaf acute (vs. obtuse) apices and hyaline (vs. greenish) margins and median leaf bases oblique (vs. truncate) and from *S. vernicosa* by the median leaf upper surfaces made up of quadrangular and rectangular (vs. undistinguishable, somewhat appearing quadrangular or elongate) papillate cells and lateral leaf margins on acroscopic side serrate to denticulate (vs. short-ciliate, at least along proximal ⅔) and on basiscopic side sparingly denticulate or entire to slightly denticulate distally (vs. short-ciliate at least along proximal ⅓).

#### Type.

Venezuela – Brazil. Venezuelan, Brazilian frontier, Planicie de Zuluaga, Río Titirico, 2300 m, 10–15 Oct 1970, *J.A. Steyermark 103872* (holotype: NY; isotypes: MO, PMA).

#### Description.

*Plants* terrestrial, epiphytic or epipetric. *Stems* creeping, stramineous, 10–20 cm long, 0.4–0.8 mm diam., non-articulate, often flagelliform, not stoloniferous, 1–2(3)-branched. *Rhizophores* axillary along proximal ½ of stem, filiform, 0.1 to 0.2 mm diam. *Leaves* dimorphic, coriaceous. *Lateral leaves* distant to imbricate towards stem and branch apices, ascending, broadly deltate-ovate, broadly ovate or ovate-elliptic, (1.0)1.3–1.7 × 0.8–1.7 mm; bases rounded, acroscopic bases overlapping the stem, basiscopic bases free from the stem; margins hyaline, made up of a band 2–4 cells wide, the cells elongate and papillate, parallel to margin, papillae in a single row over cell lumen, acroscopic margins serrate to denticulate, basiscopic margins sparingly denticulate or entire to slightly denticulate distally; apices broadly acute to acute, tipped by 2–4 teeth; both surfaces without idioblasts, upper surfaces mostly glabrous but with short hairs on submarginal and marginal sections of basiscopic half of the lamina, made up of quadrangular to rectangular, irregularly-walled, papillate or not cells, papillae 5–10 over cell lumen arranged in 2 or 3 rows, midribs not prominent, without stomata, lower surfaces glabrous, made up of elongate or asymmetric, straight or slightly sinuate-walled, papillate cells, papillae 8–20 over cell lumen arranged in 2 or 3 rows, midribs raised and prominent at mid-distal section of lamina, stomata concentrated on raised portion of midribs. *Median leaves* distant to imbricate towards stem and branch tips, ascending and, on branches, they may arise at ca. 45° angle, ovate to ovate-deltate, 1.0–1.5 × 0.6–1.0 mm; bases oblique, inner bases truncate to rounded, outer bases ventricose and often tufted with 2–8 very short hair or teeth; inner margins narrowly hyaline, made up of a band 1 or 2 cells wide, the cells elongate and papillate, parallel to margin, papillae in a single row over cell lumen, serrate to denticulate, outer margins greenish, denticulate at proximal ¼, otherwise sparingly denticulate or entire along middle and denticulate at distal ¼; apices acute, tipped by 2–4 teeth; both surfaces without idioblasts, upper surfaces mostly glabrous but occasionally with short hairs near marginal, submarginal and submedial section of outer half and on submedial section of inner half near distal ¼, made up of quadrangular or with some rectangular, irregularly-walled, papillate cells, papillae 5–10 over cell lumen arranged in 2 or 3 rows, midribs raised and prominent or bevelled specially at mid-distal section of each laminae, stomata concentrated on raised portion of midribs and some marginal on lower ½ of outer margins, lower surfaces glabrous, made up of elongate or asymmetric, straight or slightly sinuate-walled cells, midribs not prominent. *Axillary leaves* similar to lateral leaves. *Strobili* terminal and single on branch tips, quadrangular, 0.5–5 mm long. *Sporophylls* monomorphic, without a laminar flap, ovate to ovate-elliptic, 0.9–1.3 × 0.5–1.3 mm, with a keel along midrib on upper surfaces; base rounded; margins greenish, minutely denticulate to entire; apices acute; both surfaces without conspicuous idioblasts, both surfaces glabrous; *dorsal sporophylls* with upper surfaces green, except for the half that overlaps the ventral sporophylls where it is hyaline, lower surfaces greenish-hyaline; *ventral sporophylls* with both surfaces greenish-hyaline. *Megasporangia* one or two at the base of ventral rows; *megaspore* yellow, rugulate-reticulate on proximal faces with fissurate microstructure, reticulate-granular on distal faces with granulose and perforate microstructure, 410–445 μm diam. *Microsporangia* in dorsal rows and distal ¾ of ventral rows; *microspores* deep orange, proximal and distal faces not studied and not measured.

#### Habitat, distribution and phenology.

*Selaginella squamulosa* grows as a terrestrial, epiphytic or epipetric plant on open slopes to cliff base, in rills and wet depressions of swampy savannas or on scrub dominated forests at 1950–2743 m; it is known from the Guiana Highlands in Cerro de la Neblina, Amazonas State of Venezuela and in Río Cuaburí, Amazonas State of Brazil and found fertile from October through to February.

#### Etymology.

The specific epithet derives from the Latin “*squamulosus*”, meaning minutely scaly and refers to the tiny, stiff leaves that resemble small scales.

**Conservation status.** *Selaginella squamulosa* is known from two distinct localities at high elevation in isolated tepuis in the Amazon basin of Venezuela and Brazil where human presence is scarce and immediate threats have not been reported. Therefore, it is tentatively considered of Least Concern (LC) according to IUCN (2012) categories and criteria.

#### Additional specimens examined (paratypes).

**VENEZUELA**. **Amazonas**: Depto. Río Negro, Camp VII, Cerro de la Neblina, 5.1 km NE of Pico Phelps (= Neblina), 21.5 km E [of] Neblina Base Camp, ca. 2150 m, 5 Feb 1985, *Beitel 85161* (NY–2 sheets, PMA), Neblina Massif, Camp 12, 1950 m, 26–27 Feb 1985, *Boom et al. 6011* (NY, PMA, UC). **BRAZIL**. **Amazonas**: Río Negro, Río Cuaburí, 8500–9000 ft [2591–2743 m], 2 Dec 1965, *Maguire et al. 60466* (NY).

#### Discussion.

*Selaginella squamulosa* is distinguished by its centipede-like habit with slender and creeping stems, rhizophores restricted to the lower halves of the stems or often present on flagelliform stems and branches (i.e. *Steyermark 103872*, NY), coriaceous, scaly-like leaves with raised and prominent midribs on the lower and upper surfaces of the lateral and median leaves, respectively and acute leaf apices. In addition, the midribs on the upper surfaces of the median leaf are straight to strongly arcuate. In the latter case, the inner half of each leaf lamina becomes slightly wider than the outer one. It is further strikingly distinct by having the lateral leaf acroscopic halves on lower surfaces twice as wide as the basiscopic ones. Furthermore, as noted in other *Selaginella* species, on a duplicate specimen of *S. squamulosa* (i.e. *Steyermark 103872*, MO), a rhizophore becomes a leaf-bearing shoot. Remarkably, *Boom et al*. *6011* (NY, PMA, UC) has a more slender habit with the upper leaf surfaces hispidulous. Apparently, these hairs become caducous since they were not observed in a similar collection (i.e. *Beitel 85161*, NY, PMA) or in the more coriaceous form represented by the type.

The holotype and an isotype of *Selaginella squamulosa* at NY and MO, respectively, were originally identified as *S. cruegeri* Jenman vel aff., which is a synonym of *S. minima* Spring. The latter species differs most noticeably from *S. squamulosa* by its ascending (vs. creeping) habit, chartaceous (vs. coriaceous) leaves, median leaf with broadly hyaline margins (vs. inner margins narrowly hyaline and outer margins greenish) and white (vs. yellow) megaspores. A paratype of *S. squamulosa* (i.e. *Boom et al*., *6011*, NY, PMA, UC) was determined as *S. rhodostachya* Baker vel aff. Nevertheless, *S. squamulosa* is easily set aside from *S. rhodostachya* by its coriaceous (vs. chartaceous) leaves, oblique (vs. rounded to truncate) median leaf bases with the outer bases ventricose (vs. rounded) and ovate (vs. ovate-elliptic) lateral leaf with its acroscopic margins, as well as both margins of the median leaf serrate to denticulate (vs. often long-ciliate). Another of the paratypes of *S. squamulosa* (i.e. *Maguire et al. 60466*, NY) was determined as *S. brachyclada* Baker. However, it differs from the latter by having the inner and outer margins of the median leaf narrowly hyaline and greenish, respectively (vs. both margins widely hyaline) and serrate to denticulate (vs. faintly denticulate), oblique (vs. truncate) bases, ovate or ovate-elliptic (vs. ovate-cordate) lateral leaf, which is distinctly wider at the middle (vs. at the base), ascending (vs. spreading) and deep yellow (vs. white to light-yellow) megaspores that lack (vs. have) a distinct equatorial flange.

*Selaginella squamulosa* is part of the “*Selaginella vernicosa* group” and, amongst these, it is morphologically close to *S. psittacorhyncha* and *S. vernicosa*. It differs from these two species by the characters discussed under the diagnosis and by its median leaf with the midribs raised at the distal ½ of the laminae but not properly extending into the apices (vs. extending into the apices) with the upper surfaces occasionally puberulent (vs. glabrous). *Selaginella squamulosa* differs from *S. arrecta* by its median and lateral leaf apices broadly acute to acute (vs. long-acuminate to aristate), median leaf inner margins narrowly (vs. widely) hyaline, which consists of 1 or 2 (vs. 3–5) elongate cells wide and ca. 10 stomata on raised midribs (vs. up to 85 stomata, widespread on central portion of lamina). In addition, in *S. squamulosa*, 2–4 teeth tip the acute apices, whereas the acuminate or aristate apices of *S. arrecta* are made of 6–10, narrowly elongate and papillate cells. *Selaginella squamulosa* differs from *S. roraimensis*, *S. marahuacae* and *S. scalariformis* by its narrowly ovate-deltate or ovate (vs. ovate-oblong or oblong in *S. roraimensis* and ovate-elliptic in *S. marahuacae* and *S. scalariformis*) lateral leaves with midribs on lower surfaces distinctly raised (vs. plane in profile). *Selaginella squamulosa* is further separated from *S. roraimensis* by the lateral leaf basiscopic margins extended (vs. slightly reflexed), from *S. marahuacae* by its acute (vs. shortly acuminate) median leaf apices and from *S. scalariformis* by its distant to imbricate and ascending (vs. distant and patent) lateral leaves.

### Combination and status novo

#### Selaginella philipsonii

Taxon classificationPlantaeSelaginellalesSelaginellaceae

(Jermy & Rankin) Valdespino, comb. et stat. nov.

urn:lsid:ipni.org:names:77173692-1

Figure 11

Selaginella philipsonii (Jermy & Rankin) Valdespino≡Selaginella ovifolia Baker, subsp. philipsonii Jermy & Rankin, Bull. Brit. Mus. (Nat.Hist.) 9(4): 294. 1981. Type: Colombia. Meta / Vaupés: Sierra de la Macarena, Río Guapaya, 03°38'50.72"N, 72°50'45.05"W, 450 m, 29 Nov 1949, *W.R. Philipson, J.M. Idrobo & A. Fernández 1607a* (holotype: BM barcode BM000905679!).

##### Description.

*Plants* terrestrial or epipetric. *Stems* creeping, stramineous, 6–10 cm long, 0.2 to 0.3 mm diam., non-articulate, not flagelliform, not stoloniferous, 1-branched. *Rhizophores* axillary and dorsal throughout the stems, filiform, 0.05–0.1 mm diam. *Leaves* dimorphic, chartaceous. *Lateral leaves* imbricate, ascending to spreading, ovate, ovate-elliptic or ovate-oblong, (1.0)1.2–1.7 × 0.6–1.2 mm; bases rounded, acroscopic bases overlapping the stem, basiscopic bases free from the stem; acroscopic margins on upper surfaces greenish to greenish hyaline, made up of a band 1 or 2 cells wide, the cells elongate and papillate, parallel to margins, the papillae in one or two rows over each cell lumen, on lower surfaces hyaline, made up of a band 3–5 cells wide, the cells elongate and papillate, parallel to margin, the papillae in one or two rows over each cell lumen, long-ciliate throughout, the hairs long on proximal ¾ and distally smaller, basiscopic margins on upper and lower surfaces greenish, made up of a band 1 or 2 cells wide, the cells elongate and glabrous, parallel to margins, sparsely short-ciliate throughout or seemingly denticulate along proximal ¾ and with short hairs along distal ⅓ and with marginal stomata along proximal ½; apices obtuse to broadly acute or acute, tipped by (1) 2 (4), often divergent, long cilia; upper surfaces with elongate, idioblast-like cells at the base, the idioblasts papillate, the papillae in two rows, otherwise idioblast absent, most of the surface made up of irregular, quadrangular to rectangular, irregularly-walled, papillate cells, the papillae 9–15 irregularly arranged over each cell lumen or some cells glabrous, especially toward basiscopic bases, the laminae pubescent near basiscopic margins, the midrib not prominent, without stomata, the lower surfaces glabrous, made up of elongate, straight-walled, idioblast-like and papillate cells, the papillae 10–30 over each cell lumen, arranged in 2 or 3 rows and often interconnected, midrib not raised or prominent, with stomata in 2 or 3 rows concentrated along raised portion of midrib. *Median leaves* imbricate, ascending, narrowly lanceolate to narrowly lanceolate-elliptic, 0.5–1.2 × 0.3–0.6 mm; bases oblique to obtuse, glabrous; the inner margins broadly hyaline, made up of a band 2–7 cells wide, the cells elongate, idioblast-like, papillate and parallel to margins, papillae in 1 or 2 rows over each cell lumen and some of these interconnected, proximally entire and short-ciliate along distal ⅔, the outer margins greenish, made up of 2 or 3 elongate and glabrous cells and with the submarginal portion of the lamina conspicuously hyaline, made up of a band of 8–12 cells wide, the cells elongate, idioblast-like, papillate and parallel to the margins, papillae in 1 or 2 rows over each cell lumen and some of these interconnected; apices acute, attenuate to shortly acuminate, tipped by 1–5 long, divergent cilia or more often by two long, divergent cilia, the acumen or arista 0.1–0.3 mm long; both surfaces without idioblasts, except for the outer margins that have similar hyaline and papillate cells as the inner margins and submarginal portion of outer margins on the upper surfaces, the upper surfaces glabrous, made up of similar cells as the upper surfaces of the lateral leaves, the midrib not prominent and difficult to observe, stomata few, on a single row along distal ½ of each lamina, the lower surfaces glabrous, made up of elongate, straight-walled and papillate cells, papillae 10–30 over each cell lumen, arranged in 2 or 3 rows and often interconnected, midrib on lower surfaces not raised or prominent, stomata absent. *Axillary leaves* absent or, if seemingly present, corresponding to lateral leaves. *Strobili* terminal and single on branch and stem tips, quadrangular, 1.5–6.0 mm long. *Sporophylls* monomorphic, without a laminar flap, ovate-lanceolate, 0.8–1.2 × 0.5–0.6 mm, with a well-developed, puberulent to shortly ciliate keel along midribs on upper surfaces; bases rounded; margins hyaline, dentate to minutely ciliate; apices acuminate, the acumen 0.1–0.3 mm long; both surfaces without conspicuous idioblasts, glabrous; *dorsal sporophylls* with upper surfaces green, except for the halves that overlap the ventral sporophylls where they are hyaline, lower surfaces hyaline; *ventral sporophylls* with both surfaces hyaline. *Megasporangia* along distal ⅛ of ventral and dorsal rows; *megaspore* light- to deep yellow, proximal and distal faces smooth to slightly rugulate, the microstructure not observed, 180–200 μm diam. *Microsporangia* on ventral and dorsal rows along proximal ⅜; *microspores* deep orange, the ornamentation not observed, not measured.

**Figure 11.**
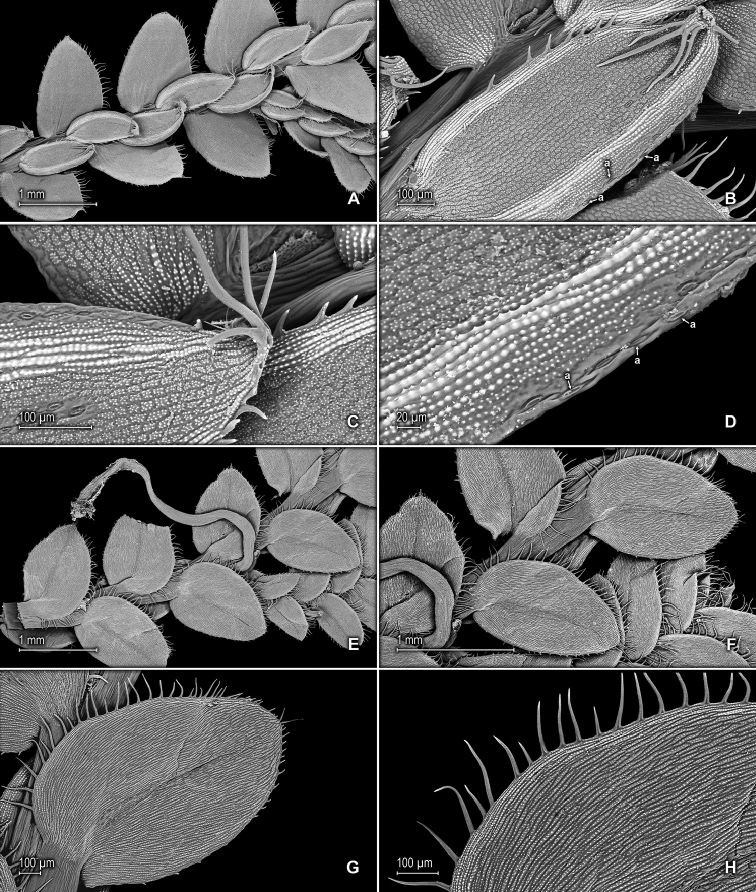
*Selaginella philipsonii* (Jermy & Rankin) Valdespino. **A** Section of upper surface of stem showing median and lateral leaves **B** Close-up of median leaf, upper surfaces; note, inner margin and submarginal portion of outer margin composed of elongate, idioblast-like and papillate cells and stomata (a) along outer margin **C** Close-up of median leaf distal portion, upper surface; note, apex tipped by long, divergent cilia, cells with papillae and stomata along outer margin **D** Close-up of median leaf, upper surface; note, marginal stomata (a), submarginal portion of outer margin composed of elongate, idioblast-like and papillate cells and lumen of lamina cells with multiple papillae **E** Section of lower surface of stem showing lateral leaves, outer halves of median leaves and rhizophore **F** Close-up of (**E**) **G** Close-up of lateral leaf, lower surface (one of the leaf shown in **E**) **H** Close-up of lateral leaf, lower surface (same leaf shown in **G**); note, stomata along midrib, elongate, idioblast-like and papillate cells and long cilia along acroscopic margin. **A–H** taken from *Steyermark & Rabe 96652* (GH).

##### Habitat, distribution and phenology.

*Selaginella philipsonii* grows on soil, wet slopes, wet rocks along riverbanks or on shaded bluffs; 100–600 m; it is known from the Meta Department of Colombia and in the State of Mérida, Venezuela and found fertile from February through to November.

##### Conservation Status.

*Selaginella philipsonii* was sporadically collected from 1949 through to 1981 in the Amazon basin of Colombia and Venezuela. No recent collections were seen. This could be, however, due to its being a minute and easily overlooked species. Consequently, based on the lack of reliable information to assign a conservation status, it is considered Data Deficient (DD), according to IUCN (2012) categories and criteria.

##### Additional specimens examined.

**COLOMBIA**. **Casanare**: Tauramena, 600 m, 13 Apr 1963, *Uribe Uribe 4290* (US). **Guaviare**: San José del Guariare [Guaviare], *s.d.*, *Schultes 11120* (GH). **Meta**: Caño Piedra de Candela, 5 km from Remolino, 13 Feb 1969, *Pinto & Sastre 841* (COL, P), Río Guayabero, 10 km below Caño Lozada, 350 m, 18 Jan 1959, *Pinto & Bischler 239* (COL [digital image]). **Vaupés**: Angostura No. 2, Río Guayabero, 23 Feb 1969, *Pinto & Sastre 1024* (COL). **VENEZUELA**. **Mérida**: on slope above dam site on Río Caparo, 31 km ESE of Santa Barbara, 07°41'N, 71°28'W, 100–250 m, 9 Mar 1980, *Liesner & González 9263* (F, PMA); 2–4 km above dam site on Río Guaimaral, 07°45'N, 71°29'W, 200–400 m, 16 Mar 1981, *Liesner & González 10648a* (MO). **Tachira**: W of Ayarí, 200 m, 21 Aug 1966, *Steyermark & Rabe 96652* (GH), along old highway, W of Ayarí, 07°32'N, 71°53'W, 250 m, 7 Nov 1979, *Steyermark et al*. *119488* (MO, UC).

##### Discussion.

*Selaginella philipsonii* was originally described as a subspecies of *S. ovifolia* by Jermy and Rankin in Alston et al. (1981: 294). They indicated, however, that Alston considered it a new species, a conclusion with which the author fully concurs and hence its recognition here at that level. According to Alston (1955: 244), *S. ovifolia* is a taxon found in the Dominican Republic, Haiti, Jamaica, Puerto Rico and Belize. Certainly, these two taxa share a moss-like habit and median leaf apices ending in long cilia. Nevertheless, *S. philipsonii* is most distinguished by the median leaves on main stems imbricate, oblong-elliptic with the inner margins hyaline, made up of a band of 2–7, elongate and papillate cells and shortly ciliate and the outer margins greenish, made up of 2 or 3 elongate and glabrous cells and the submarginal portion of the laminae conspicuously hyaline, composed of a band of 8–12 cells wide, with the cells elongate and papillate, parallel to the margins, the papillae in 1 or 2 rows over each cell lumen and some of these interconnected (see Figs 11A–D, 16B in Alston et al. 1981: 296), entire and the apices acute, attenuate to shortly acuminate, tipped by 1–5 (Fig. 11C) or often 2 long, divergent cilia, the acumen or arista 0.1–0.3 mm long. It is further characterized by the lateral leaves acroscopic margins long-ciliate, the basiscopic margins denticulate, the apices obtuse to broadly acute or acute, tipped by (1) 2 (4), often divergent, long cilia (Fig. 11E–G). In addition, SEM images of the median leaves show stomata along the outer margins, as well as on the midribs (Fig. 11B–D). Conversely, *S. ovifolia* is characterized by median leaves on main stems distant, elliptic to ovate-elliptic, the margins sparingly short- to long-ciliate, having the outer margins and the outer submarginal portion of the laminae greenish, composed of rounded cells or the outer margins only slightly hyaline on distal ¼ and made up of only 1 or 2 elongate and papillate cells, while the inner margins are greenish, comprised of roundish cells, the apices long-acuminate, tipped by 2 long cilia, the lateral leaf acroscopic margins long-ciliate, while the basiscopic margins are short- to long-ciliate and the apices acute or short- to long-acuminate, tipped by 1–3 teeth or short cilia.

Jermy and Rankin in Alston et al. (1981: 294) cited two specimens from Norte de Santander: Catatumbo, Campo Oru, 08°30'30"N, 73°15'52"W, 350–500 m, 13 May 1959, *Bischler 2397* (COL!) and Campo Tibú, Río Tibú, 08°28'19"N, 72°55'08"W, 200 m, 16 May 1959, *Bischler 2493* (COL!) as S. ovifolia subsp. philipsonii. These specimens, however, have soboliferous stems, median leaves narrowly elliptic to ovate-lanceolate, 0.3–0.6 × 0.15–0.3 mm with the inner and outer margins dentate to shortly ciliate, the outer margins and the submarginal portion of the laminae greenish, made of rounded cells or the outer margins only slightly hyaline composed of only 1–3 elongate cells and puberulent bases with 1–3 stiff, short hairs or tooth-like projections. Therefore, the author believes it is best to exclude those specimens from *S. philipsonii*.

*Selaginella philipsonii* is morphologically close and may be confused with *S. homaliae* A. Braun and *S. schultesii* Alston ex Crabbe & Jermy, which are both from South America, because of their moss-like habit, slender rhizophores, minute leaves and keeled sporophylls with the keel short-ciliate to dentate. *Selaginella philipsonii* differs noticeably from *S. homaliae* and *S. schultesii* by its median leaf outer margins greenish (vs. conspicuously hyaline) with the submarginal portion of the laminae conspicuously hyaline (vs. greenish), which is made up of a band of 8–12 elongate and papillate (vs. composed of roundish and glabrous) cells that are parallel to the margins, the apices acute, attenuate to shortly acuminate, tipped by 1–5 (Fig. 11C) or often 2 long, divergent cilia (vs. apices aristate, denticulate with the tip gradually tapering into a long arista and tipped by 1–3 teeth in *S. schultesii* and mucronate to shortly acuminate and tipped by 1 or 2 teeth in *S. homaliae*) and the outer margins entire (vs. long-ciliate in *S. schultesii* and short-ciliate in *S. homaliae*). *Selaginella philipsonii* is set aside further from *S. homaliae* by ovate or ovate-elliptic (vs. oblong) lateral leaves with the leaf apices obtuse to broadly acute or acute, tipped by (1) 2 (4), often divergent, long cilia (vs. truncate to obtuse, not tipped by cilia) and axillary and dorsal (vs. axillary) rhizophores.

*Selaginella valdepilosa* Baker is another South American moss-like species that could be confused with *S. philipsonii*; however, the former has the lateral leaves broadly ovate-orbicular or broadly ovate-elliptic with the basiscopic bases distinctly, albeit sparsely, short- to long-ciliate and the median leaves broadly ovate-orbicular to ovate-deltate with both margins distinctly long-ciliate and the outer bases noticeably knobbed with the submarginal portion composed of round cells.

*Selaginella philipsonii* stands out from other moss-like taxa such as *S. achotalensis* Shelton & Caluff, *S. apoda* (L.) C. Morren, *S. armata* Baker, *S. cristalensis* Shelton & Caluff, *S. eatonii* Hieron. ex Small [syn.: S. armata var. eatonii (Hieron. ex Small) B.F. Hansen & Wunderlin, *S. bracei* Hieron. ex Small], *S. ludoviciana* A. Braun, *S. orbicularifolia* Shelton & Caluff, *S. prasina* Baker (syn.: *S. undata* Shelton & Caluff) and *S. rotundifolia* Spring by its main stems median leaves imbricate, oblong-elliptic and widely hyaline submarginal portion of the laminae, as well as lateral leaves long-ciliate along acroscopic margins. In keeping with Valdespino (1993) by following Buck (1978), Buck and Lucanski (1976) and Somers and Buck (1975) and contrary to Hansen and Wunderlin (2000), *S. eatonii* is considered a different species from *S. armata* as is *S. ludoviciana* from *S. apoda*. Finally, following Valdespino et al. (2015) *S. undata* is considered a synonym of *S. prasina*.

## Supplementary Material

XML Treatment for Selaginella altheae

XML Treatment for Selaginella squamulosa

XML Treatment for Selaginella philipsonii
